# Benign splenic lesions in BAP1-tumor predisposition syndrome: a case series

**DOI:** 10.1038/s41431-024-01623-w

**Published:** 2024-06-01

**Authors:** Joao Miranda, Priya Dave, Yelena Kemel, Rania Sheikh, Grace Zong, Lina Posada Calderon, Marie Will, Ying L. Liu, Michael Walsh, Zsofia K. Stadler, Kenneth Offit, Alicia Latham, Diana Mandelker, Ying-Bei Chen, Pamela Causa Andrieu, Maria Isabel Carlo

**Affiliations:** 1 Department of Radiology, Memorial Sloan Kettering Cancer Center, New York, NY USA; 2 Department of Urology, Montefiore Medical Center, Albert Einstein College of Medicine, Bronx, NY USA; 3 Sloan Kettering Institute, Memorial Sloan Kettering Cancer Center, New York, NY USA; 4 Department of Medicine, Memorial Sloan Kettering Cancer Center, New York, NY USA; 5 Department of Urology, New York Presbyterian Hospital, Weill Cornell Medicine, New York, NY USA; 6 Department of Pediatrics, Memorial Sloan Kettering Cancer Center, New York, NY USA; 7 Department of Pathology & Laboratory Medicine, Memorial Sloan Kettering Cancer Center, New York, NY USA; 8 Department of Radiology, Mayo Clinic, Rochester, MN USA

**Keywords:** Cancer genetics, Cancer genetics

## Abstract

BAP1-Tumor Predisposition Syndrome (TPDS) is caused by germline variants in *BAP1* and predisposes to solid tumors. After observation of a radiologically malignant-appearing splenic mass with benign pathology in a patient with BAP1-TPDS, we sought to retrospectively characterize splenic lesions in individuals with BAP1-TPDS seen at a comprehensive cancer center. A dedicated radiology review for splenic abnormalities was performed. We identified 37 individuals with BAP1-TPDS, 81% with a history of cancer. Of 33 individuals with abdominal imaging, 10 (30%) had splenic lesions, and none were shown to be malignant on follow-up. Splenectomy in an individual with suspected splenic angiosarcoma showed a benign vascular neoplasm with loss of nuclear staining for BAP1 in a subset of cells. Benign splenic lesions appear to be common and potentially BAP1-driven in individuals with BAP1-TPDS; confirmation of these findings could lead to more conservative management and avoidance of splenectomy.

## Introduction

BAP1-Tumor Predisposition Syndrome (BAP1-TPDS) is an autosomal dominant disorder caused by variants in the *BAP1* gene. *BAP1*, located in chromosome 3p, is a tumor suppressor gene that encodes a ubiquitin carboxy-terminal hydrolase. Through its deubiquitinase activity, it plays an important functional role in post-translational modification of proteins that regulate cellular processes, such as DNA repair, cellular proliferation and differentiation, and cell cycle [1]. BAP1-TPDS is characterized by increased risk of solid tumors, including mesothelioma, cutaneous and uveal melanoma, renal cell carcinoma, and BAP1-inactivated melanocytic tumors [2].

Larger sequencing studies in unselected cancer individuals show that BAP1-TPDS may be more common than previously thought, but the limited number of families reported to date still limits our understanding of the phenotypic spectrum [3]. Given the recent identification of this syndrome, long-term follow-up data on tumor screening outcomes are also lacking. Notably, in our clinical practice, we observed an individual with BAP1-TPDS with a suspected splenic angiosarcoma on imaging, which on splenectomy showed a benign vascular neoplasm with loss of nuclear BAP1 staining in a subpopulation of lesional cells. This prompted us to investigate this observation in a systematic manner. Here, we describe the prevalence and characteristics of splenic lesions in individuals with BAP1-TPDS followed at a comprehensive cancer center.

## Materials and methods

### Patients and radiologic and pathologic evaluation

We retrospectively identified individuals who had genetic evaluation at our institution and were found to have likely pathogenic or pathogenic variants in *BAP1* (*n* = 37). Clinical characteristics were abstracted from the 37 electronic medical record (EMR); from 33 patients who had abdominal imagining available, further radiological analysis was conducted. For those with available cross-sectional abdominal imaging (computed tomography (CT), magnetic resonance imaging (MRI) or positron emission tomography (PET-CT)) available, two radiologists (PCA, JM) analyzed the images in consensus by independently reviewing and interpreting each image, engaging in a collaborative discussion to reconcile discrepancies, and reaching a mutual agreement on the final interpretation. Therefore, the interreader agreement was not estimated. For available pathologic samples, BAP1 immunohistochemistry was performed using a monoclonal antibody (Clone C-4, Santa Cruz, TX). This study was approved by the Memorial Sloan Kettering Cancer Center Institutional Review Board.

## Results

**Fig. 1:**
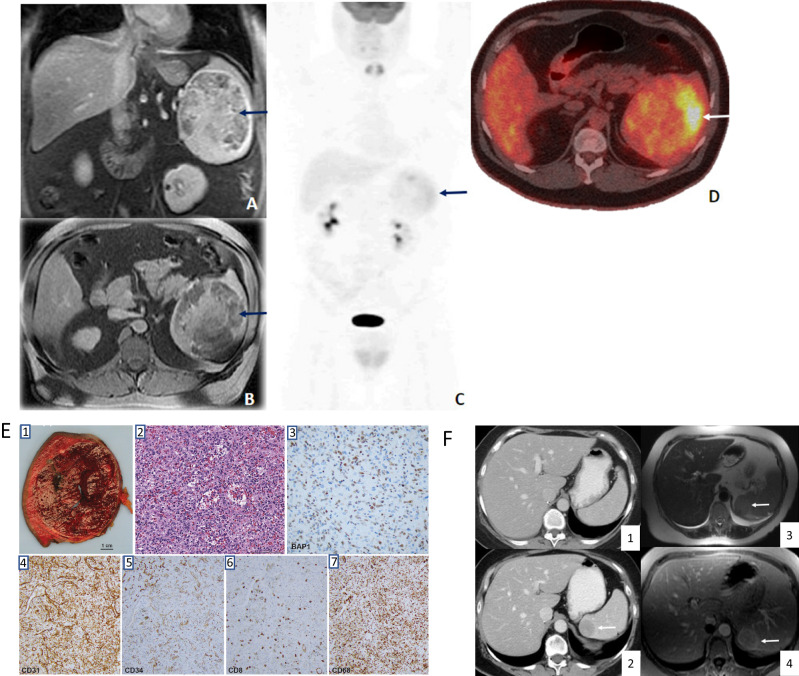
Radiographic and pathologic examples of benign splenic lesions in patients with BAP1 germline variants. **A**–**D** MRI and PET imaging in a patient with radiological features suggestive of a malignant splenic lesion, favoring angiosarcoma. **E** Gross and microscopic images of the resected splenic vascular lesions seen in images (**A**–**D**). **F** CT and MRI images of another patient with well-defined and isodense splenic lesion after 6 years of follow-up.

We identified 37 individuals with likely pathogenic or pathogenic variants in *BAP1* from 35 families; most individuals (81.1%) had a history of cancer (Supplemental Table 1). Of 33 individuals with abdominal imaging available for review, 9 (27%) had splenic lesions (Table 1). In total, 146 image studies were reviewed. Specifically, 25 individuals had CT scans, 7 had MRI scans, and 1 had a PET-CT scan on baseline. Splenic lesions had a range of image characteristics; the majority (90%) were consistent with benign findings. Three cases had well defined, vascular lesions characterized by vascular channels with similar signal intensity (MRI) or density (CT) to the spleen and showing progressive homogeneous contrast enhancement, which are features indicative of sclerosing angiomatoid nodular transformation (SANT) or littoral cell angioma, with an example in Fig. 1F. Additionally, three cases exhibited small subcentimeter hypodense lesions in the spleen, likely benign in nature, such as cysts or hemangiomas. One individual showed well-circumscribed lesion on imaging, with no or minimal contrast enhancement, suggesting probably hamartoma. One case had a minimally complex cystic lesion with mildly thickened septae, suggesting a lymphangioma. Most (70%) showed image stability during the follow-up period (median 46.3 months). In the three cases with new or increased lesions, the growths did not exhibit the aggressive patterns typically associated with malignancies.

**Table 1:** Radiographic Characteristics of Splenic Lesions.

Study ID	Patient Age at Analysis	Personal Cancer History	Baseline Modality (CT/MRI)	Radiologic Features	Splenectomy	Pathology	Vascular Lesion	Lesion Stability^a^	Follow up time (months)	
MSK_BAP1_8	65	Yes	CT	Few subcentimeter hypodense lesions, probably benign such as cysts or hemangiomas		No	Yes	No	19.6	
MSK_BAP1_10	64	Yes	CT	Few subcentimeter hypodense lesions, probably benign such as cysts or hemangiomas		No	Yes	No	23.3	
MSK_BAP1_13	83	Yes	CT	Few subcentimeter hypodense lesions, probably benign such as cysts or hemangiomas		No	Yes	No	29	
MSK_BAP1_18	53	Yes	CT	Minimally complex cystic lesion with mildly thickened septae, 1.8 × 1.4 cm, probably lymphangioma		No	Yes	Yes	12.4	
MSK_BAP1_24	59	Yes	CT	Well-defined, homogeneous mass with distinct borders and low vascularity, probably hamartoma, measuring up to 10 cm		No	No	Yes	10.3	
MSK_BAP1_27	68	Yes	CT	Well-defined and isodense lesion with progressive homogeneous contrast enhancement, 7.4 × 6.4 cm. It probably represents benign vascular in etiology such as SANT or littoral cell angioma		No	Yes	Yes	99.8	
MSK_BAP1_28	59	Yes	CT	Well-defined and isodense lesion with progressive homogeneous contrast enhancement, 9.0 × 7.8 cm. It probably represents benign vascular in etiology such as SANT or littoral cell angioma		No	Yes	Yes	12.3	
MSK_BAP1_29	48	No	MRI	Heterogenous mass with vascular channels, progressive enhancement on MRI, and increased FDG avidity on PET - imaging would favor angiosarcoma, 10.6 ×9.5 cm.	Yes	Yes	Yes	NA	60.3	
MSK_BAP1_35	75	Yes	MRI	Well-defined & isodense lesion with vascular channels, progressive homogeneous contrast enhancement, probably benign vascular in etiology such as SANT or littoral cell angioma, 3.3 × 2.5 cm.	Yes	Fine needle aspiration consistent with granuloma but subsequent splenectomy sample not available for review	Yes	Yes	149.6	

^a^Lesion stability refers to the lack of significant changes or growth observed.

*CT* computed tomography, *FDG* fluorodeoxyglucose, *MRI* magnetic resonance imaging, *NA* not applicable, SANT sclerosing angiomatoid nodular transformation.

Four individuals had a splenectomy; two for splenic lesions suspicious for primary malignancy, one as part of their routine cancer surgery and one for unknown reasons. One case was suspicious for angiosarcoma (patient ID 29) on imaging since it demonstrated marked enhancement on MRI and marked FDG uptake on PET-CT (Fig. 1A–D). Pathology showed a solitary 11 cm benign vascular neoplasm involving the splenic red pulp (Fig. 1E). Immunostains revealed the tumor cells to be positive for CD31, CD68, CD163, and CD34 (focal), whereas CD8 and CD 21 were negative. This immunoprofile was not typical for either hemangioma or littoral cell angioma. Immunohistochemistry for BAP1 showed loss of nuclear staining in a subset of cells in the vascular lesion (Fig. 1E3). A second individual (patient ID 35) (Fig. 1F) had a fine needle aspirate of the splenic vascular lesion, with epithelioid histiocytes and inflammation without malignancy. The patient still had a splenectomy at an outside institution. The pathology slides were not available for our review, but the report noted “hemorrhagic infarct with areas of older fibrosis.”

## Discussion

We describe the novel finding of a high prevalence of benign, mostly vascular splenic lesions in individuals with BAP1-TPDS, with pathology suggesting a role for BAP1 in its etiology. Splenic lesions were observed in 30% of individuals with available abdominal imaging and followed at a comprehensive cancer center. This contrasts with the rare nature of incidental splenic lesions, which have been reported in only 1–3% of routine imaging [4–6]. Despite most of the individuals in our cohort having a history of cancer, none of the lesions were pathologically confirmed to be malignant, and when followed longer term, none were suspicious for primary malignancy or metastatic disease.

Solid splenic masses rarely occur in routine clinical practice and present a diagnostic challenge. These lesions are sometimes discovered incidentally during imaging studies performed for unrelated reasons and are usually benign lesions [4]. Benign solid splenic lesions include hemangiomas, hamartomas, and granulomas [7–9], while rarer entities such as SANT/littoral cell angioma, inflammatory pseudotumor, or Gaucher nodules may be seen [10, 11]. Our study involving individuals with BAP1-TPDS noted a diverse spectrum of splenic lesions, including cysts/hemangiomas, hamartomas, and granulomas. However, the most prevalent lesions were consistent with SANT/ littoral cell angioma.

Malignant solid splenic lesions, including lymphoma, leukemic infiltrates, metastases, and rarely, angiosarcoma [12–15] can be observed. In our study, one case radiographically suspicious for angiosarcoma, also exhibited marked FDG uptake, but on pathology was consistent with a benign vascular proliferation. Interestingly, BAP1 staining on IHC showed loss of nuclear staining in some of the cells in the vascular lesion.

Pre-clinical data suggests a role for BAP1 in splenic growth and myeloproliferative disease. In mouse models, conditional deletion of *BAP1* in hematopoietic cells led to a fully penetrant myeloproliferative disease with universal splenomegaly [16]. We hypothesize that loss of BAP1 function in human splenic littoral cells causes proliferation through mechanisms that are currently unknown. Interestingly, to date, there has been no reported increased risk of myeloid or lymphoid malignancies in BAP1-TPDS, and the mechanism by which *BAP1* germline individuals may increase the risk of benign vascular lesions but not lead to malignancy needs to be further explored.

Currently, National Comprehensive Cancer Network (NCCN) guidelines recommend cross-sectional abdominal imaging every 2 years for patients with BAP1-TPDS [17]. The knowledge that benign vascular splenic lesions are part of the BAP1-TPDS spectrum could have important implications for clinical management. It could lead to more conservative management of these lesions and avoidance of splenectomy, which can lead to serious complications such as increased risk of severe infections. Although these are relatively rare procedures in the population, in our cohort, four (11%) individuals had undergone splenectomies, with benign findings, underscoring the potential clinical benefit in the BAP1-TPDS population of conservative management. A limitation of our study is its observational nature and lack of control group to clarify the rate of benign splenic lesions in patients without a genetic predisposition. Furthermore, even if there is this increased incidence, it is still unclear whether the *BAP1* germline mutation is a direct cause, or whether it may be another association with cancer or its treatment [18, 19]. As more patients are diagnosed and screened for BAP1-TPDS, these findings will need to be confirmed in other cohorts.

In summary, the novel description of benign splenic lesions in a significant proportion of our BAP1-TPDS cohort, exceeding the expected prevalence in the general population, highlights the importance of further investigation of the BAP1-TPDS. Understanding these splenic lesions’ prevalence, characteristics, and potential etiology is crucial for establishing appropriate surveillance protocols and clinical management strategies for individuals with BAP1-TPDS.

### Supplementary information

Supplementary Table 1 and 2

## Data Availability

The data supporting this article are provided in the supplementary files available in the online version of this article at the publisher’s website.
