# Supplementary material for: Benign splenic lesions in BAP1-tumor predisposition syndrome: a case series
Source: Eur J Hum Genet. 2024 Jun 1;32(8):1027–31. doi: 10.1038/s41431-024-01623-w (PMC11291683; doi:10.1038/s41431-024-01623-w)
Supplement: Supplementary file 1 — Supplementary Table 1 and 2 [file 41431_2024_1623_MOESM1_ESM.docx]

**Supplemental Table 1.** Patient characteristics

|  | N=37 (100%) |
| --- | --- |
| Age at time of analysis [years] |  |
| Median | 54 |
| Range | 7 - 83 |
| Sex assigned at birth |  |
| Female | 21 (53.8) |
| Race/Ethnicity |  |
| White | 29 (78%) |
| Black | 4 (10%) |
| Asian | 2 (5%) |
| N/A | 2 (5%) |
| History of cancer |  |
| Yes | 30 (81.1%) |
| No | 7 (18.9%) |
| Cancer Types* |  |
| Kidney | 8 (24.2%) |
| Mesothelioma | 8 (24.2%) |
| Melanoma | 10 (12.1%) |
| Ovary | 3 (9.1%) |
| Bile duct | 3 (9.1%) |
| Uveal melanoma | 1 (3.0%) |
| Liver | 1 (3.0%) |
| Breast | 1 (3.0%) |
| Bladder | 1 (3.0%) |
| Colorectal | 1 (3.0%) |
| Non-small cell lung cancer | 1 (3.0%) |
| Miscellaneous brain tumor | 1 (3.0%) |

* Some patients have history of multiple tumors

**Supplemental Table 2.** *BAP1* Likely Pathogenic or Pathogenic Variants

| Case ID # | Variant | Variation | MSK Classification |
| --- | --- | --- | --- |
| MSK_BAP1_1 | c.1833_1834delGA | NM_004656.4:c.1833_1834delGA | Pathogenic |
| MSK_BAP1_2 | c.442G>T | NM_004656.4:c.442G>T | Pathogenic |
| MSK_BAP1_3 | c.437G>A | NM_004656.4:c.437G>A | Likely Pathogenic |
| MSK_BAP1_4 | c.1203dupT | NM_004656.4:c.1203dupT | Pathogenic |
| MSK_BAP1_5 | c.437+1G>T Intron 6 | NM_004656.4:c.437+1G>T | Pathogenic |
| MSK_BAP1_6 | c.295G>A | NM_004656.4:c.295G>A | Likely Pathogenic |
| MSK_BAP1_7 | c. 1983+1_1983+3delinsAT |  | Likely Pathogenic |
| MSK_BAP1_8 | c.1777C>T | NM_004656.4:c.1777C>T | Pathogenic |
| MSK_BAP1_9 | c.580+1G>A | NM_004656.4:c.580+1G>A | Likely Pathogenic |
| MSK_BAP1_10 | c.375+202_455dup (Intragenic Duplication) |  | Likely Pathogenic |
| MSK_BAP1_11 | c.1675_1684delACAGGCCTGC |  | Pathogenic |
| MSK_BAP1_12 | c. 1110dupC | NM_004656.4:c.1110dupC | Pathogenic |
| MSK_BAP1_13 | Deletion exons 15-17 |  | Pathogenic |
| MSK_BAP1_14 | c.774_783+6delTCTGCAGCAGGTAGGT |  | Likely Pathogenic |
| MSK_BAP1_15 | c.784-1G>A | NM_004656.4:c.784-1G>A | Likely Pathogenic |
| MSK_BAP1_16 | c.295G>A | NM_004656.4:c.295G>A | Likely Pathogenic |
| MSK_BAP1_18 | c.956C>G | NM_004656.4:c.956C>G | Likely Pathogenic |
| MSK_BAP1_19 | c.2012delA | NM_004656.4:c.2012del | Likely Pathogenic |
| MSK_BAP1_20 | c.437+1G>A | NM_004656.4:c.437+1G>A | NA |
| MSK_BAP1_22 | Whole Gene Deletion |  | Pathogenic |
| MSK_BAP1_23 | c.1378_1379delTC | NM_004656.4:c.1378_1379delTC | Likely Pathogenic |
| MSK_BAP1_24 | c.256-2A>G | NM_004656.4:c.256-2A>G | Likely Pathogenic |
| MSK_BAP1_25 | c.935delG |  |  |
| MSK_BAP1_26 | c.1778dupA |  |  |
| MSK_BAP1_27 | c.375+1G>A | NM_004656.4:c.375+1G>A | Likely Pathogenic |
| MSK_BAP1_28 | c.375+1G>A | NM_004656.4:c.375+1G>A | Likely Pathogenic |
| MSK_BAP1_29 | c.21delG |  | Pathogenic |
| MSK_BAP1_30 | c.1203T>G | NM_004656.4:c.1203T>G | Pathogenic |
| MSK_BAP1_31 | c.1777C>T | NM_004656.4:c.1777C>T | Pathogenic |
| MSK_BAP1_32 | c.1993C>T | NM_004656.4:c.1993C>T | Pathogenic |
| MSK_BAP1_33 | c.463A>T | NM_004656.4:c.463A>T | Pathogenic |
| MSK_BAP1_34 | c.639dupT |  | Pathogenic |
| MSK_BAP1_35 | c.1254T>A | NM_004656.4:c.1254T>A | Pathogenic |
| MSK_BAP1_36 | c.580+1G>A | NM_004656.4:c.580+1G>A | Likely Pathogenic |
| MSK_BAP1_37 | c.580+1G>A | NM_004656.4:c.580+1G>A | Likely Pathogenic |
| MSK_BAP1_38 | Deletion Exons 15-17, |  | Pathogenic |
| MSK_BAP1_39 | c.2T>C | NM_004656.4:c.2T>C | Pathogenic |

*MSK=Memorial Sloan Kettering;
